# Considering the utility of urinary amino acids for early identification of non-diabetic chronic kidney disease

**DOI:** 10.1093/ckj/sfaf198

**Published:** 2025-06-20

**Authors:** Henry H L Wu, David Cantor, Fei Chi, Long The Nguyen, Rajkumar Chinnadurai, Carol A Pollock, Sonia Saad

**Affiliations:** Renal Research, Kolling Institute of Medical Research, Royal North Shore Hospital & The University of Sydney, Sydney, Australia; Department of Renal Medicine, Royal North Shore Hospital, Northern Sydney Local Health District, Sydney, Australia; Macquarie Analytical and Fabrication Facility, Australian Proteome Analysis Facility, Macquarie University, Sydney, Australia; Macquarie Analytical and Fabrication Facility, Australian Proteome Analysis Facility, Macquarie University, Sydney, Australia; Renal Research, Kolling Institute of Medical Research, Royal North Shore Hospital & The University of Sydney, Sydney, Australia; Faculty of Biology, Medicine & Health, The University of Manchester, Manchester, UK; Renal Research, Kolling Institute of Medical Research, Royal North Shore Hospital & The University of Sydney, Sydney, Australia; Department of Renal Medicine, Royal North Shore Hospital, Northern Sydney Local Health District, Sydney, Australia; Renal Research, Kolling Institute of Medical Research, Royal North Shore Hospital & The University of Sydney, Sydney, Australia

To the Editor,

The excess excretion of amino acids in urine signifies loss of nutrients and metabolic dysregulation, which are pathophysiological processes involved in chronic kidney disease (CKD). Urinary amino acids have previously been investigated as a novel non-invasive metabolomic biomarker for early identification of diabetic kidney disease (DKD) [1–3]. However, the utility of urinary amino acids has not been studied in the context of non-diabetic early CKD, which is also a prevalent CKD population at risk of adverse clinical outcomes.

Our group conducted a cross-sectional pilot study, performing urinary amino acid profiling in 20 adult non-diabetic patients (Table 1). All 20 patients had kidney biopsy performed and CKD status was determined here as per interstitial fibrosis and tubular atrophy (IFTA) grading. There were 10 patients with IFTA 0%–10% (i.e. no CKD) and 10 patients with IFTA 10%–25% (i.e. early CKD). The two groups were statistically similar in age, a median age 67 years (interquartile range (IQR) 41–71 years) in the no CKD group versus 62 years (IQR 57–68 years) in the early CKD group, *P *= .880; sex, all 20 patients were male; and estimated glomerular filtration rate (eGFR), a median eGFR 90 ml/min/1.73 m^2^ (IQR 90–90 ml/min/1.73 m^2^) in the no CKD group versus 88 ml/min/1.73 m^2^ (IQR 86–90 ml/min/1.73 m^2^) in the early CKD group, *P *= .246. In the early CKD group, there were seven patients with micro- or macroalbuminuria. Urine samples were collected immediately before kidney biopsy and processed through HPLC and mass. Specific metabolites were identified, and urinary amino acid concentration (reflecting absolute abundance) and mole% (reflecting relative abundance) were determined via bioinformatics analyses. The differences in median urinary amino acid concentration (in µg/ml) and mole% between the non-diabetic early CKD and no CKD groups were examined.

**Table 1:** Information on age (years), eGFR, albuminuria status, co-morbidities, treatments received at the timepoint of kidney biopsy, and diagnosis following kidney biopsy for the 20 study patients.

Patient ID	Age (years)	eGFR (ml/min/1.73 m^2^)	Albuminuria status	Co-morbidities	Treatments received at the timepoint of kidney biopsy	Diagnosis following histological evaluation of kidney biopsy tissue
IFTA 0 (*n* = 10)						
1	31	90	Nil	Asthma	Budesonide, Salbutamol	Nil kidney disease: minimal glomerular sclerosis and IFTA noted
2	73	90	Nil	Osteoarthritis	Paracetamol, Voltaren emulgel	Nil kidney disease: very minimal glomerular sclerosis noted
3	71	90	Nil	Hypercholesterolemia	Atorvastatin	Nil kidney disease
4	48	90	Nil	Hypertension Trigeminal neuralgia	Indapamide, Losartan, Carbamazepine	Nil kidney disease: very minimal hypertensive nephrosclerotic changes noted
5	68	90	Nil	Nil	Nil	Nil kidney disease
6	77	90	Nil	Hypercholesterolemia	Simvastatin	Nil kidney disease: minimal glomerular sclerosis and IFTA noted
7	41	90	Nil	Nil	Nil	Nil kidney disease
8	66	90	Nil	Rheumatoid arthritis	Methotrexate, Folic acid, Paracetamol, Ibuprofen	Nil kidney disease
9	73	90	Nil	Hypercholesterolemia	Rosuvastatin	Nil kidney disease: minimal glomerular sclerosis and IFTA noted
10	68	90	Nil	Hypertension	Indapamide	Nil kidney disease: very minimal hypertensive nephrosclerotic changes noted
IFTA 1 (*n* = 10)						
11	79	90	Micro	Osteoarthritis	Paracetamol, Voltaren emulgel	Non-steroidal anti-inflammatory drug-induced kidney tubulointerstitial fibrosis
12	68	88	Micro	Hypertension	Indapamide, Losartan, Prazosin	Hypertensive nephropathy
13	64	90	Micro	Hypertension Hypercholesterolemia	Indapamide, Losartan, Rosuvastatin	Hypertensive nephropathy
14	61	90	Nil	Nil	Nil	Immunoglobulin A nephropathy
15	48	88	Micro	Hypertension	Indapamide, Losartan	Hypertensive nephropathy
16	67	87	Micro	Hypertension	Indapamide, Losartan	Immunoglobulin A nephropathy
17	57	90	Nil	Hypercholesterolemia	Rosuvastatin	Early CKD changes due to high cholesterol
18	47	90	Micro	Hypertension	Indapamide	Hypertensive nephropathy
19	73	82	Macro	Hypertension Hypercholesterolemia	Indapamide, Losartan, Atorvastatin	Minimal change disease and hypertensive nephropathy
20	58	85	Nil	Hypertension	Prazosin	Immunoglobulin A nephropathy

Eighteen amino acids were identified and characterized across the non-diabetic early CKD and no CKD groups (Table 2). The median urinary concentration (3.6 vs 0.8 µg/ml, *P *= .012) and mole% (1.18% vs 0.69%, *P *= .034) of valine and the median urinary mole% of alanine (6.95% vs 5.12%, *P *= .049) were significantly higher in the non-diabetic early CKD compared to the no CKD group. Conversely, the median urinary mole% of histidine (11.2% vs 15.3%, *P *= .041) was significantly lower in the non-diabetic early CKD compared to the no CKD group.

**Table 2:** Comparison of urinary amino acid concentration (µg/ml) and urinary amino acid mole% between the non-diabetic early CKD and no CKD groups.

	Urinary amino acid concentration (µg/ml) expressed as median (interquartile range)			Urinary amino acid mole% expressed as median (interquartile range)		
	Early CKD (*n* = 10)	No CKD (*n* = 10)	*P*	Early CKD (*n* = 10)	No CKD (*n* = 10)	*P*
Histidine	51.6 (19.8–77.5)	59.4 (34.5–100.8)	.290	11.2 (9.24–15.4)	15.3 (14.8–15.8)	.041
Asparagine	13.2 (7.6–15.7)	6.8 (4.4–19.3)	.450	3.25 (2.71–3.71)	2.55 (2–2.88)	.070
Taurine	38 (33–70)	61 (50–115)	.096	18.5 (9.92–23)	25.7 (21.8–28)	.131
Serine	25.6 (12–33.6)	20.3 (13.5–36.4)	.940	8.05 (7.68–9.74)	7.65 (7.24–8.83)	.364
Glutamine	36.5 (23–50)	35.6 (23–50)	.650	9.49 (8.36–10.8)	10.4 (9.8–10.6)	.290
Arginine	2.1 (1.1–6.1)	2.1 (1.1–4)	.705	0.55 (0.48–0.99)	0.46 (0.33–1.02)	.597
Glycine	44.5 (19.7–89)	37 (17–78)	.364	22.8 (18.6–30.4)	17.3 (14.9–24.4)	.082
Threonine	11.2 (1–15.1)	10 (5.4–22.7)	.940	3.36 (3.18–3.73)	3.10 (2.77–3.86)	.762
Alanine	16.8 (8.4–30)	15.3 (8–20)	.364	6.95 (5.25–9.31)	5.12 (5.06–6.28)	.049
Cystine	8.3 (2.6–11)	6.6 (3.5–8.7)	.364	0.97 (0.79–1.47)	0.94 (0.75–0.96)	.450
Lysine	7.1 (4–19)	8 (5.8–29.7)	.364	2.04 (1.4–2.92)	2.70 (1.61–6.60)	.496
Tyrosine	5.2 (3.6–12.6)	8 (3.3–13.2)	.940	1.44 (1.2–2.11)	1.51 (1.23–1.66)	.705
Methionine	1.38 (0–2.45)	1.31 (0–2.1)	.850	0.32 (0–0.40)	0.38 (0.25–0.48)	.343
Valine	3.6 (1.9–4.8)	0.8 (0–1.29)	.012	1.18 (0.87–1.52)	0.69 (0–1.04)	.034
Isoleucine	0.95 (0–1.41)	0.8 (0–1.3)	.699	0.26 (0–0.33)	0.19 (0–0.26)	.122
Leucine	2.44 (1.61–3.7)	1.3 (1.1–1.97)	.545	0.77 (0.51–1.06)	0.66 (0.61–0.76)	.496
Phenylalanine	3.5 (1.86–7.6)	3.2 (2–5.33)	.650	0.96 (0.77–1.22)	0.90 (0.64–1.09)	.326
Tryptophan	5.6 (3.4–10.7)	7.17 (2.9–11.1)	.821	1.37 (1.22–1.55)	1.24 (0.99–1.44)	.406

Previous studies observed significant associations between urinary levels of valine and DKD progression [1, 3]. The exact mechanisms on how valine, a branched-chain amino acid (BCAA), contributes to DKD pathophysiology is still being investigated for both Type 1 and 2 diabetes mellitus (DM). It is now widely considered that increased BCAA levels is associated with insulin deficiency, resistance, and dysregulation in glucose metabolism, all risk factors for DKD development [4]. Otherwise, urinary alanine levels are found to be predictive of DKD [2]. The complex interplay between alanine, its associated enzyme alanine aminotransferase, and CKD/DKD is not yet fully explored, but nonetheless deemed to be potentially correlated with mechanisms involving mitochondrial dysfunction and oxidative stress [5]. Decreased plasma and urinary levels of histidine were observed in patients with DKD compared to DM patients and healthy controls [3]. Linked to protein synthesis as well as anti-oxidation and anti-inflammation, significant associations between decreased histidine levels and protein-energy wasting, oxidative stress and inflammation in CKD/DKD, and adverse CKD/DKD outcomes were established [6].

In summary, our pilot study demonstrated by using HPLC-MS that urinary levels of specific amino acids differed between individuals with non-diabetic early CKD and no CKD. There is potential in utilizing the urinary levels of valine, alanine, and histidine as non-invasive biomarkers for early identification of non-diabetic CKD. Further delineation on the roles of these amino acids in non-diabetic CKD pathophysiology is needed going forward.
